# Synthesis, Structural Elucidation, and *In Vitro* Antitumor Activities of Some Pyrazolopyrimidines and Schiff Bases Derived from 5-Amino-3-(arylamino)-1*H*-pyrazole-4-carboxamides

**DOI:** 10.3797/scipharm.1211-07

**Published:** 2013-01-03

**Authors:** Taghrid S. Hafez, Souad A. Osman, Hisham Abdallah A. Yosef, Amira S. Abd El-All, Ashraf S. Hassan, Abdallah A. El-Sawy, Mohamed M. Abdallah, Mahmoud Youns

**Affiliations:** 1 Department of Organometallic and Organometalloid Chemistry, National Research Centre, El-Behoos Street, Dokki, 12622 Cairo, Egypt.; 2 Department of Chemistry Natural and Microbial products, National Research Centre, El-Behoos Street, Dokki, 12622 Cairo, Egypt.; 3 Chemistry Department, Faculty of Science, Benha University, Benha, Egypt.; 4 Univet Pharmaceuticals Ltd, Balteem, Egypt.; 5 Biochemistry and Molecular Biology Department, Faculty of Pharmacy, Helwan University, Cairo, Egypt.

**Keywords:** 5-Aminopyrazole-4-carboxamides, Pyrazolo[1,5-*a*]pyrimidines, Schiff bases, Antitumor agents, Ferrocenecarboxaldehyde

## Abstract

The reaction of 5-amino-3-(arylamino)-1*H*-pyrazole-4-carboxamides **1a,b** with acetylacetone **2** and arylidenemalononitriles **5a–c** yielded the pyrazolo[1,5-*a*]-pyrimidine derivatives **4a,b** and **7a–f** respectively. On the other hand, Schiff bases **9a,b** and **12a–j** were obtained upon treatment of carboxamides **1a,b** with isatin **8** and some selected aldehydes **11a–e**. The newly synthesized compounds were characterized by analytical and spectroscopic data. Representative examples of the synthesized products **4a,b, 7e, 7f, 9b, 12b–f, 12h,** and **12j** were screened for their *in vitro* antitumor activities against different human cancer cell lines and the structure-activity relationship (SAR) was discussed.

## Introduction

Of the various human diseases, cancer, human immunodeficiency virus (HIV), and hepatitis C virus (HCV) are the major scourges of humanity. Therefore, the identification of novel, potent, selective, less toxic anticancer and antiviral agents remains one of the most pressing health problems. A literature survey revealed that pyrazolo[1,5-*a*]pyrimidines are purine analogues which are well-known for their importance in biological applications as antimetabolites [1]. They act as potent inhibitors of the enzymes HCV polymerase [2], checkpoint kinase 1 (CHK1) [3], cyclin-dependent kinase 2 (CDK2) [4], and c-Src kinase [5]. Moreover, pyrazolo[1,5-*a*]pyrimidine derivatives have significant antimicrobial [6], antitumor [7], and anti-inflammatory [8] activities.

On the other hand, Schiff bases are important classes of compounds in the medicinal field, which have biological applications including antimicrobial [9], antioxidant [10, 11], and antitumor [12–15] activities.

In view of these facts, we report herein the synthesis of a new series of substituted pyrazolo[1,5-*a*]pyrimidines and Schiff bases derived from 5-aminopyrazole derivatives for the examination of their antitumor activity.

## Results and Discussion

### Chemistry

The starting compounds, 5-amino-3-(phenylamino)-1*H*-pyrazole-4-carboxamide (**1a**) [16] and 5-amino-3-[(4-methoxyphenyl)amino]-1*H*-pyrazole-4-carboxamide **1b** were utilized in preparing the target compounds (Schemes 1 and 2).

Pyrazole derivative **1b** was obtained by the reaction of 2-cyano-3-[(4-methoxy– phenyl)amino]-3-(methylthio)acrylamide with hydrazine hydrate in boiling ethanol in the presence of triethylamine. Its structure was assigned upon compatible elemental analyses and spectral data. Correct analytical data and molecular weight determination (MS) corresponded to C_11_H_13_N_5_O_2_ (*m/z*=247 [M^+^]). The IR spectrum (KBr/cm^−1^) revealed the presence of strong absorption bands at 3439, 3277, and 3163 corresponding to −NH_2_ and −NH. Bands at 1659 and 1624 were attributed to C=O and C=N group frequencies, respectively, and the band at 1558 was due to C=C (aromatic). The ^1^H NMR spectrum (DMSO-d_6_, δ ppm) revealed the presence of signals at 3.64 for −OCH_3_ protons as a singlet, four singlets at 5.74, 6.60, 8.68, and 10.82 assigned for two −NH_2_ and two −NH protons, which were D_2_O exchangeable. The two doublets present at 6.75 (2H) and 7.24 (2H) were assigned for the four aromatic protons (AB system, *J_HH_=8.4* Hz), and its ^13^C NMR spectrum (DMSO-d_6_, δ ppm) showed signals at 55.6 (−OCH_3_), 86.2 (C_4_, pyrazole), 114.6 (2C, aromatic), 117.4 (2C, aromatic), 137.0 (C, aromatic), 148.2 (C_5_, pyrazole), 152.2 (C_3_, pyrazole), 152.8 (C, aromatic), and 167.2 (C=O, amide).

Compounds **1a,b** were reacted with acetylacetone **2** in boiling glacial acetic acid to afford the corresponding new pyrazolo[1,5-*a*]pyrimidines **4a,b** (Scheme 1). The structures of **4a,b** were confirmed on the basis of their analytical and spectral data. Compound **4a,** taken as a representative example, revealed the molecular formula C_15_H_15_N_5_O (m/z=281 [M^+^]), and its IR spectrum (KBr/cm^−1^) showed strong absorption bands at 3374 and 3142, corresponding to −NH_2_ and −NH, a band at 1658 due to C=O, two bands at 1626 and 1596 for C=N, and a band at 1562 due to C=C (aromatic). Its ^1^H NMR spectrum (DMSO-d_6_, δ ppm) showed two singlets at 2.50 and 2.66 due to two −CH_3_ group protons and a signal at 6.93 due to the H-6 proton of the pyrimidine nucleus. Two singlets present at 7.47 and 9.57 were assigned for the −NH_2_ and −NH protons, which were D_2_O exchangeable. Two triplets present at 6.90 (1H) and 7.30 (2H) were assigned for the three aromatic protons and one doublet at 7.66 (2H) for the two aromatic protons (*J_HH_=7.65* Hz), and their ^13^C NMR spectrum (DMSO-d_6_, δ ppm) showed signals at 17.1 (−CH_3_), 24.6 (−CH_3_), 86.9 (C_3_, pyrazolopyrimidine), 109.3 (C_6_, pyrazolopyrimidine), 117.5 (2C, aromatic), 121.2 (C, aromatic), 129.5 (2C, aromatic), 140.8 (C_3a_, pyrazolopyrimidine), 146.4 (C, aromatic), 146.7 (C_7_, pyrazolopyrimidine), 156.7 (C_2_, pyrazolopyrimidine), 161.3 (C_5_, pyrazolopyrimidine), and 166.4 (C=O, amide).

The formation of compounds **4a,b** was therefore assumed to proceed *via* initial attack of the exocyclic amino group of **1** on the keto group of the 1,3-dicarbonyl compound **2** followed by intramolecular cyclization *via* elimination of water.

Arylidenemalononitriles **5a–c** were reacted with **1a,b** in ethanol under reflux conditions to give 7-amino-5-aryl-2-(arylamino)-6-cyanopyrazolo[1,5-*a*]pyrimidine-3-carboxamides **7a–f** (Scheme 1). The structures of **7a–f** were established based on their analytical and spectral data. Thus, as an example, the mass spectrum of compound **7d** showed an ion peak at m/z 397, which corresponded to [M^+^−2H], and its IR spectrum (KBr/cm^−1^) showed bands at 3399, 3333, and 3151 for −NH_2_ and −NH, 2211 for C≡N, 1646 for C=O, 1597 for C=N, and 1509 for C=C (aromatic) groups. Its ^1^H NMR spectrum (DMSO-d_6_, δ ppm) revealed the presence of a singlet at 3.71 corresponding to protons of the −OCH_3_ group, three singlets at 7.58, 8.92, and 9.43 due to two −NH_2_ and −NH protons which were D_2_O exchangeable. Two doublets at 7.75 (2H) and 7.85 (2H) were assigned for four aromatic protons (*J_HH_=8.4 Hz*). A multiplet appeared at 6.86–7.55 for five aromatic protons and its ^13^C NMR spectrum (DMSO-d_6_, δ ppm) showed signals at 55.6 (−OCH_3_), 74.9 (C_6_, pyrazolopyrimidine), 89.6 (C_3_, pyrazolopyrimidine), 114.7 (2C, aromatic), 116.7 (C≡N), 119.3 (2C, aromatic), 128.8 (2C, aromatic), 129.0 (C, aromatic), 129.2 (2C, aromatic), 131.1 (C_3a_, pyrazolopyrimidine), 133.9 (C, aromatic), 137.3 (C, aromatic), 149.8 (C, aromatic), 154.2 (C_2_, pyrazolopyrimidine), 156.9 (C_5_, pyrazolopyrimidine), 161.6 (C_7_, pyrazolopyrimidine), and 166.2 (C=O, amide).

The formation of compounds **7a–f** was assumed to proceed *via* initial attack of the exocyclic amino function of the compounds **1a,b** on the α,β-unsaturated system in compound **5,** followed by intramolecular cyclization and spontaneous autooxidation through the loss of the H_2_ molecule [17] (Scheme 1).

Condensation of **1a**,**b** with isatin **8** in boiling ethanol gave 3-(arylamino)-5-[(2-oxoindolin-3-ylidene)amino]-1*H*-pyrazole-4-carboxamides **9a,b** in excellent yields (Scheme 2). Structures of compounds **9a,b** were confirmed on the basis of elemental analysis and spectral data. As an example, the IR spectrum (KBr/cm^−1^) of **9a** showed strong stretching bands at 3427, 3317, and 3181 for –NH_2_ and −NH, two strong absorption bands at 1687 and 1623 due to two C=O groups, and a band at 1597 for the C=N group. Its ^1^H NMR spectrum (DMSO-d_6_, δ ppm) showed a diffused multiplet at 6.89–7.53 due to aromatic and −NH_2_ protons. Three singlets appeared at 9.14, 10.95, and 12.98 due to three −NH protons, which were D_2_O exchangeable.

A trial for ring closures of **9a,b** to give the spiro derivatives **10a,b** was unsuccessful, even after their prolonged boiling in glacial acetic acid.

Schiff bases **12a–j** were obtained by the reaction of 1*H*-pyrazolo-4-carboxamides **1a,b** with some selected aldehydes **11a–e** [e.g. aromatic- **11a–c**, heteroaromatic- **11d,** and ferrocenecarboxaldehyde **11e**] in boiling ethanol using a catalytic amount of triethylamine (Scheme 2).

The structures of compounds **12a–j** were confirmed on the basis of their analytical and spectral data. As an example, the mass spectrum of compound **12e** exhibited a molecular ion peak at *m*/*z* 413 (C_21_H_19_FeN_5_O), which was also the base peak. Its IR spectrum (KBr/cm^−1^) showed stretching bands at 3359 and 3168 for −NH_2_ and −NH, as well as bands at 1660, 1588, and 1563 for C=O, C=N, and C=C (aromatic) groups, respectively. Its ^1^H NMR spectrum (DMSO-d_6_, δ ppm) showed the 5H of the unsubstituted ferrocene ring at 4.29 as a singlet, the 4H of the monosubstituted ferrocene ring at 4.68 (2H) and 4.88 (2H) as two singlets, and a signal at 9.03 due to the 1H of the −N=CH– group, three singlets at 7.50, 8.72, and 12.55 due to the −NH_2_ and two −NH protons which were D_2_O exchangeable, and a multiplet at 6.79–7.28 for five aromatic protons. The ^13^C NMR spectrum (DMSO-d_6_, δ ppm) of **12e** showed signals at 70.2 (5C, ferrocene ring), 73.3 (4C, ferrocenyl ring), 79.1 (C, ferrocenyl ring), 92.8 (C_4_, pyrazole), 116.6 (2C, aromatic), 119.8 (C, aromatic), 129.5 (2C, aromatic), 142.0 (C, aromatic), 148.3 (−N=CH−), 153.2 (C_3_ & C_5_, pyrazole), and 167.0 (C=O, amide).

### Biological evaluation

#### In vitro antitumor screening

Preliminary experiments were done to check the availability of the prepared compounds as antitumor agents. We selected different varieties of the newly synthesized compounds containing variable groups and then we evaluated their *in vitro* cytotoxic activities against the human breast cancer cell line (MCF7) where Doxorubicin was used as a standard drug [18]. The results were expressed as the IC_50_ value, which corresponds to the concentration required for 50% inhibition of cell growth of the treated cells when compared to that of control cells.

From the results in Table 1, it was found that the IC_50_ values of compounds **7f**, **12j,** and **12e** were 0.085 μM, 9.294 μM, and 28.48 μM, respectively, which exhibited the highest cytotoxic activities, followed by compound **4a** (IC_50_=122.9 μM) which also showed better activity than the reference drug Doxorubicin (IC_50_=96.41 μM), while compound **12d** (IC_50_=280.0 μM) showed lower activity than the reference drug.

The promising results obtained from screening against the MCF7 tumor cell line (Table 1) encouraged us to study the cytotoxicity of the tested compounds by using the MTT assay against different human cancer cell lines, including: cervical carcinoma (KB), ovarial carcinoma (SK OV-3), CNS cancer (SF-268), non-small cell lung cancer (NCl H460), colonadenocarcinoma (RKOP 27) (Table 2), anti-leukemia (HL60, U937, K562), melanoma (SK-MEL-28), and neuroblastoma (GOTO, NB-1) (Table 3). The cytotoxic effects of the tested compounds over the cell lines of HeLa (cervical), HT1080 (fibrosarcoma), and HepG2 (liver) were also investigated (Table 4).

Screening the cytotoxicity of the tested compounds on cervical carcinoma (KB), where Fluorouracil was used as a standard drug (IC_50_=4.46 nM), showed that the seven tested compounds **7e, 12b–f,** and **12j** were more potent than the standard. The most potent one was **12e** (IC_50_=0.54 nM).

On the ovarial carcinoma (SK OV-3) cell line, compounds **7e, 12b, 12d, 12e, 12f,** and **12h** (IC_50_=0.30, 0.44, 3.30, 0.32, 0.90, and 2.20 nM respectively) were more potent than the standard drug Doxorubicin (IC_50_=4.16 nM). The most potent one was found to be **7e** (IC_50_=0.30 nM).

Studying the effects of cytotoxicity for the tested compounds on the CNS cancer (SF-268) cell line, using Cytarabine (IC_50_=7.68 nM) as a standard drug, revealed that compounds **4a, 7e,** 7**f, 12c, 12d, 12e, 12f**, **12h,** and **12j** were more active than the standard drug, where **12e** (IC_50_=0.30 nM) was the most promising one.

On the non-small cell lung cancer (NCl H460) cell line, the tested compounds, except for **12e** and **12h** (IC_50_=6.60 & 7.00 nM respectively), were more potent than the standard drug Gencitabine hydrochloride (IC_50_=2.13 nM).

On the colonadenocarcinoma (RKOP 27) cell line, the tested compounds, except for **12c, 12d, 12e,** and **12h,** were found to be less active than the standard drug Capecitabine (IC_50_=4.33 nM). Compounds **4b** and **12j** had a comparable activity to Capecitabine.

The study of the cytotoxicity on the leukemia (HL60) cell line indicated that compounds **4b** (IC_50_=7.50 nM), **7f** (IC_50_=3.57 nM), **9b** (IC_50_=5.30 nM), **12h** (IC_50_=5.50 nM), and **12j** (IC_50_=5.60 nM) were less potent than Doxorubicin (IC_50_=1.13 nM).

On the leukemia (U937) cell line, compound **12d** (IC_50_=0.09 nM) was the most potent, but **7f** was the least bioactive (IC_50_=55.0 nM).

On the leukemia (K562) cell line, compound **7f** (IC_50_=0.17 nM) was the most potent among the tested compounds, followed by compound **12e** (IC_50_=0.43 nM), but compound **7e** (IC_50_=8.00 nM) was less potent than Doxorubicin (IC_50_=6.66 nM).

From the estimation of the cytotoxicity on the melanoma (SK-MEL-28) cell line, the tested compounds were less active than the standard drug Aldesleukin (IC_50_=3.45 nM), except **12b** (IC_50_=3.20 nM) was slightly more active.

On the neuroblastoma (GOTO) and (NB-1) cell lines, compound **12d** was the most potent (IC_50_=0.45 nM) and (IC_50_=0.64 nM), respectively, among the tested compounds. Moreover, it was more active than the standard drug Doxorubicin (IC_50_=4.73 nM and IC_50_=5.15 nM, respectively).

The cytotoxicity of the tested compounds on the HeLa (cervical) cell line showed that Tamoxifen (IC_50_=0.11 nM), the standard drug used, was more active than all of the tested compounds.

On the HT1080 (fibrosarcoma) cell line, compound **12d** (IC_50_=0.54 nM) was the most potent one, but compound **12c** (IC_50_=8.97 nM) was found to be less potent than Tamoxifen (IC_50_=1.16 nM).

The cytotoxicity of the tested compounds on the HepG2 (liver) cell line showed that seven of the tested compounds were more bioactive than Tamoxifen (IC_50_=1.31 nM) in a decreasing order of **12e**>**12d**>**12j**>**12c**>**12f**>**7e**>**12h.**

Based on these results, it is evident that there is a structure-activity relationship (SAR). Shown in Table 4, from the screening of the tested compounds against the HepG2 (liver) cell line, it was found that some derivatives, in which the amino group on the pyrazole ring is linked to a phenyl group, were more active than their respective analogues with a 4-methoxyphenyl group on that nitrogen atom. Thus, compounds **12c** (IC_50_=0.66 nM) and **12e** (IC_50_=0.09 nM) were found to be more potent than **12h** (IC_50_=0.99 nM) and **12j** (IC_50_=0.62 nM), respectively. However, compounds **4a** and **4b** were found to be of a comparable potency.

On the other hand, the investigation confirmed the prominent biological activity of the ferrocenyl moiety over other substituents, where among the tested Schiff bases, compound **12e** was found to be the most potent against the HepG2 (liver) cell line. The order of activity was **12e** (IC_50_=0.09 nM)>**12d** (IC_50_=0.35 nM)>**12c** (IC_50_=0.66 nM). Similarly, **12j** (IC_50_=0.62 nM) was more active than **12h** (IC_50_=0.99 nM).

## Experimental

### Chemistry

All melting points were measured on a Gallenkamp melting point apparatus and were uncorrected. The IR spectra were recorded (KBr disk) on a Perkin Elmer 1650 FT-IR instrument. The ^1^H NMR (500 MHz) and ^13^C NMR (125 MHz) spectra were recorded on a Varian spectrometer using DMSO-d_6_ as a solvent and TMS as an internal standard. Chemical shifts were reported in ppm. Mass spectra were recorded on a Varian MAT 112 spectrometer at 70 eV. Elemental analyses were obtained from The Microanalytical Data Center at Cairo University, Egypt.

Progress of the reactions was monitored by thin-layer chromatography (TLC) using aluminum sheets coated with silica gel F_254_ (Merck), viewed by short-wavelength UV lamp detection. All evaporations were carried out under reduced pressure at 40 °C.

### 5-Amino-3-(arylamino)-1H-pyrazole-4-carboxamides (1a,b)

A mixture of cyanoacetamide (0.01 mol) and 4-(methoxyphenyl)isothiocyanate or phenylisothiocyanate (0.01 mol) was heated for 5–10 min in ethanol (25 mL) containing potassium hydroxide (0.01 mol). After cooling, methyl iodide (0.01 mol) was added. The reaction mixture was stirred at room temperature for 1 h then poured onto ice-water. The precipitated product [3-(4-arylamino)-2-cyano-3-(methylthio)acrylamide] was filtered off and recrystallized from ethanol, then its mixture with hydrazine hydrate (0.01 mol) was refluxed for 4 h in ethanol (30 mL) containing triethylamine as a catalyst. After evaporating the solvent under reduced pressure, the resulting solid product was collected by filtration and recrystallized from ethanol.

#### 5-Amino-3-(phenylamino)-1H-pyrazole-4-carboxamide (**1a**)

Yield: 80%, white crystals, m.p. 179 °C [16].

#### 5-Amino-3-[(4-methoxyphenyl)amino]-1H-pyrazole-4-carboxamide (**1b**)

Yield: 82%, white crystals, m.p. 200 °C. IR (KBr) ν_max_/cm^−1^ 3439, 3277, 3163 (NH, NH_2_), 1659 (C=O), 1624 (C=N), 1558 (C=C, aromatic). ^1^H NMR (DMSO-d_6_, δ ppm) 3.64 (s, 3H, OCH_3_), 5.74 (s, 2H, NH_2_, D_2_O exchangeable), 6.60 (s, 2H, NH_2_, D_2_O exchangeable), 6.75 (d, 2H, aromatic, *AB-system, J_HH_=8.4* Hz), 7.24 (d, 2H, aromatic, *AB-system, J_HH_=8.4* Hz), 8.68 (s, 1H, NH, D_2_O exchangeable), 10.82 (s, 1H, NH, D_2_O exchangeable). ^13^C NMR (DMSO-d_6_, δ ppm) 55.6 (−OCH_3_), 86.2 (C_4_, pyrazole), 114.6 (2C, aromatic), 117.4 (2C, aromatic), 137.0 (C, aromatic), 148.2 (C_5_, pyrazole), 152.2 (C_3_, pyrazole), 152.8 (C, aromatic), 167.2 (C=O, amide). MS *m/z* (%): 247 (59.90) [M^+^]. Anal. Calcd. (%) for C_11_H_13_N_5_O_2_ (247.25): C, 53.43; H, 5.30; N, 28.32. Found: C, 53.35; H, 5.39; N, 28.21 %.

### Synthesis of 2-(arylamino)-5,7-dimethylpyrazolo[1,5-a]pyrimidine-3-carboxamides (4a,b)

A mixture of compound **1a** or **1b** (0.01 mol) with acetylacetone **2** (0.01 mol) in glacial acetic acid (20 mL) was refluxed for 6 h, then poured onto crushed ice and the separated solid was filtered off, dried well, and recrystallized from ethanol to afford compounds **4a,b**.

#### 5,7-Dimethyl-2-(phenylamino)pyrazolo[1,5-a]pyrimidine-3-carboxamide **(4a)**

Yield: 82%, white crystals, m.p. 275–277 °C. IR (KBr) ν_max_/cm^−1^ 3374, 3142 (NH, NH_2_), 1658 (C=O), 1626, 1596 (C=N), 1562 (C=C, aromatic). ^1^H NMR (DMSO-d_6_, δ ppm) 2.50 (s, 3H, CH_3_), 2.66 (s, 3H, CH_3_), 6.90 (t, 1H, aromatic), 6.93 (s, 1H, pyrimidine H-6), 7.30 (t, 2H, aromatic), 7.47 (s, 2H, NH_2_, D_2_O exchangeable), 7.66 (d, 2H, aromatic, *J_HH_=7.65* Hz), 9.57 (s, 1H, NH, D_2_O exchangeable). ^13^C NMR (DMSO-d_6_, δ ppm) 17.1 (−CH_3_), 24.6 (−CH_3_), 86.9 (C_3_, pyrazolopyrimidine), 109.3 (C_6_, pyrazolopyrimidine), 117.5 (2C, aromatic), 121.2 (C, aromatic), 129.5 (2C, aromatic), 140.8 (C_3a_, pyrazolopyrimidine), 146.4 (C, aromatic), 146.7 (C_7_, pyrazolopyrimidine), 156.7 (C_2_, pyrazolopyrimidine), 161.3 (C_5_, pyrazolopyrimidine), 166.4 (C=O, amide). MS m/z (%): 281 (6.59) [M^+^]. Anal. Calcd. (%) for C_15_H_15_N_5_O (281.31): C, 64.04; H, 5.37; N, 24.90. Found: C, 63.90; H, 5.45; N, 25.00 %.

#### 2-[(4-Methoxyphenyl)amino]-5,7-dimethylpyrazolo[1,5-a]pyrimidine-3-carboxamide **(4b)**

Yield: 78%, white crystals, m.p. 260–262 °C. IR (KBr) ν_max_/cm^−1^ 3364, 3159 (NH, NH_2_), 1655 (C=O), 1623, 1597 (C=N), 1564 (C=C, aromatic). ^1^H NMR (DMSO-d_6,_ δ ppm) 2.50 (s, 3H, CH_3_), 2.65 (s, 3H, CH_3_), 3.69 (s, 3H, OCH_3_), 6.89 (d, 2H, aromatic, *J_HH_=7.6* Hz), 6.90 (s, 1H, pyrimidine H-6), 7.47 (s, 2H, NH_2_, D_2_O exchangeable), 7.60 (d, 2H, aromatic, *J_HH_=7.6* Hz), 9.35 (s, 1H, NH, D_2_O exchangeable). ^13^C NMR (DMSO-d_6_, δ ppm) 17.2 (−CH_3_), 24.6 (−CH_3_), 55.7 (−OCH_3_), 86.6 (C_3_, pyrazolopyrimidine), 109.2 (C_6_, pyrazolopyrimidine), 114.7 (2C, aromatic), 117.6 (2C, aromatic), 131.5 (C_3a_, pyrazolopyrimidine), 133.8 (C, aromatic), 146.6 (C_7_, pyrazolopyrimidine), 151.3 (C, aromatic), 156.9 (C_2_, pyrazolopyrimidine), 161.4 (C_5_, pyrazolopyrimidine), 166.4 (C=O, amide). MS m/z (%): 311 (47.17) [M^+^]. Anal. Calcd. (%) for C_16_H_17_N_5_O_2_ (311.34): C, 61.72; H, 5.50; N, 22.49. Found: C, 61.83; H, 5.42; N, 22.40 %.

### Synthesis of 7-amino-5-aryl-2-(arylamino)-6-cyanopyrazolo[1,5-a]pyrimidine-3-carboxamides (7a–f)

A mixture of compound **1a** or **1b** (0.01 mol) with arylidenemalononitriles **5a–c** (0.01 mol) and a catalytic amount of triethylamine (four drops) in absolute ethanol (30 mL) was refluxed for 6 h. The solvent was concentrated under reduced pressure and the solid obtained was collected and recrystallized from ethanol to give **7a–f**.

#### 7-Amino-6-cyano-5-phenyl-2-(phenylamino)pyrazolo[1,5-a]pyrimidine-3-carboxamide **(7a)**

Yield: 78%, yellow crystals, m.p. > 300 °C. IR (KBr) ν_max_/cm^−1^ 3393, 3303, 3148 (NH, NH_2_), 2212 (C≡N), 1658 (C=O), 1593 (C=N), 1564 (C=C, aromatic). ^1^H NMR (DMSO-d_6_, δ ppm) 6.94–7.55 (m, 6H, aromatic), 7.60 (s, 2H, NH_2_, D_2_O exchangeable), 7.81 (d, 2H, aromatic, *J_HH_=7.6 Hz*), 7.86 (d, 2H, aromatic, *J_HH_=8.4 Hz*), 8.97 (s, 2H, NH_2_, D_2_O exchangeable), 9.64 (s, 1H, NH, D_2_O exchangeable). ^13^C NMR (DMSO-d_6_, δ ppm) 74.6 (C_6_, pyrazolopyrimidine), 89.9 (C_3_, pyrazolopyrimidine), 116.5 (C≡N), 118.3 (2C, aromatic), 121.4 (C, aromatic), 128.5 (2C, aromatic), 129.2 (C, aromatic), 129.5 (2C, aromatic), 130.1 (2C, aromatic), 137.3 (C, aromatic), 140.6 (C_3a_, pyrazolopyrimidine), 146.8 (C, aromatic), 150.2 (C_2_, pyrazolopyrimidine), 156.7 (C_5_, pyrazolopyrimidine), 161.9 (C_7_, pyrazolopyrimidine), 166.3 (C=O, amide). MS m/z (%): 369 (29.30) [M^+^]. Anal. Calcd. (%) for C_20_H_15_N_7_O (369.38): C, 65.03; H, 4.09; N, 26.54. Found: C, 65.17; H, 4.02; N, 26.44 %.

#### 7-Amino-6-cyano-5-(4-methoxyphenyl)-2-(phenylamino)pyrazolo[1,5-a]pyrimidine-3-carboxamide **(7b)**

Yield: 65%, yellow crystals, m.p. > 300 °C. IR (KBr) ν_max_/cm^−1^ 3440, 3379, 3315 (NH, NH_2_), 2217 (C≡N), 1662 (C=O), 1621, 1596 (C=N), 1560 (C=C, aromatic). ^1^H NMR (DMSO-d_6,_ δ ppm) 3.82 (s, 3H, OCH_3_), 6.94 (t, 1H, aromatic), 7.09 (d, 2H, aromatic, *J_HH_=8.4 Hz*), 7.29 (2H, aromatic), 7.58 (s, 2H, NH_2_, D_2_O exchangeable), 7.80 (d, 2H, aromatic, *J_HH_=8.4* Hz), 7.86 (d, 2H, aromatic, *J_HH_=8.4* Hz), 8.90 (s, 2H, NH_2_, D_2_O exchangeable), 9.63 (s, 1H, NH, D_2_O exchangeable). ^13^C NMR (DMSO-d_6_, δ ppm) 55.9 (−OCH_3_), 74.4 (C_6_, pyrazolopyrimidine), 89.6 (C_3_, pyrazolopyrimidine), 114.4 (2C, aromatic), 117.0 (C≡N), 118.0 (2C, aromatic), 121.4 (C, aromatic), 129.4 (C, aromatic), 129.5 (2C, aromatic), 130.9 (2C, aromatic), 140.4 (C_3a_, pyrazolopyrimidine), 146.4 (C, aromatic), 150.0 (C_2_, pyrazolopyrimidine), 156.8 (C, aromatic), 161.1 (C_5_, pyrazolopyrimidine), 161.8 (C_7_, pyrazolopyrimidine), 166.2 (C=O, amide). MS m/z (%): 401 (8.21) [M^+^+2H]. Anal. Calcd. (%) for C_21_H_17_N_7_O_2_ (399.41): C, 63.15; H, 4.29; N, 24.55. Found: C, 63.06; H, 4.35; N, 24.43 %.

#### 7-Amino-6-cyano-5-(naphthalen-1-yl)-2-(phenylamino)pyrazolo[1,5-a]pyrimidine-3-carboxamide **(7c)**

Yield: 65%, orange crystals, m.p. 262–264 °C. IR (KBr) ν_max_/cm^−1^ 3413, 3358, 3275, 3165 (NH, NH_2_), 2217 (C≡N), 1655 (C=O), 1593 (C=N), 1558 (C=C, aromatic). ^1^H NMR (DMSO-d_6,_ δ ppm) 6.95–8.21 (m, 14H, aromatic and NH_2_), 9.11 (s, 2H, NH_2_, D_2_O exchangeable), 9.65 (s, 1H, NH, D_2_O exchangeable). Anal. Calcd. (%) for C_24_H_17_N_7_O (419.44): C, 68.72; H, 4.09; N, 23.38. Found: C, 68.85; H, 3.97; N, 23.45 %.

#### 7-Amino-6-cyano-2-[(4-methoxyphenyl)amino]-5-phenylpyrazolo[1,5-a]pyrimidine-3-carboxamide **(7d)**

Yield: 70%, Yellow crystals, m.p. > 300 °C. IR (KBr) ν_max_/cm^−1^ 3399, 3333, 3151 (NH, NH_2_), 2211 (C≡N), 1655 (C=O), 1597 (C=N), 1509 (C=C, aromatic). ^1^H NMR (DMSO-d_6_, δ ppm) 3.71 (s, 3H, OCH_3_), 6.86–7.55 (m, 5H, aromatic), 7.58 (s, 2H, NH_2_, D_2_O exchangeable), 7.75 (d, 2H, aromatic, *J_HH_=8.4 Hz*), 7.85 (d, 2H, aromatic, *J_HH_=8.4 Hz*), 8.92 (s, 2H, NH_2_, D_2_O exchangeable), 9.43 (s, 1H, NH, D_2_O exchangeable). ^13^C NMR (DMSO-d_6_, δ ppm) 55.6 (−OCH_3_), 74.9 (C_6_, pyrazolopyrimidine), 89.6 (C_3_, pyrazolopyrimidine), 114.7 (2C, aromatic), 116.7 (C≡N), 119.3 (2C, aromatic), 128.8 (2C, aromatic), 129.0 (C, aromatic), 129.2 (2C, aromatic), 131.1 (C_3a_, pyrazolopyrimidine), 133.9 (C, aromatic), 137.3 (C, aromatic), 149.8 (C, aromatic), 154.2 (C_2_, pyrazolopyrimidine), 156.9 (C_5_, pyrazolopyrimidine), 161.6 (C_7_, pyrazolopyrimidine), 166.2 (C=O, amide). MS m/z (%): 397 (17.06) [M^+^−2H]. Anal. Calcd. (%) for C_21_H_17_N_7_O_2_ (399.41): C, 63.15; H, 4.29; N, 24.55. Found: C, 63.24; H, 4.19; N, 24.60 %.

#### 7-Amino-6-cyano-5-(4-methoxyphenyl)-2-[(4-methoxyphenyl)amino]pyrazolo[1,5-a]-pyrimidine-3-carboxamide **(7e)**

Yellow crystals, m.p. 259–261 °C, yield (73%). IR (KBr) ν_max_/cm^−1^ 3396, 3313, 3190 (NH, NH_2_), 2210 (C≡N), 1646 (C=O), 1598 (C=N), 1568 (C=C, aromatic). ^1^H NMR (DMSO-d_6_, δ ppm) 3.71 (s, 3H, OCH_3_), 3.83 (s, 3H, OCH_3_), 6.85–7.09 (m, 4H, aromatic), 7.45 (s, 2H, NH_2_, D_2_O exchangeable), 7.75–7.86 (m, 4H, aromatic), 8.83 (s, 2H, NH_2_, D_2_O exchangeable), 9.43 (s, 1H, NH, D_2_O exchangeable). ^13^C NMR (DMSO-d_6_, δ ppm) 55.6 (−2OCH_3_), 74.1 (C_6_, pyrazolopyrimidine), 89.8 (C_3_, pyrazolopyrimidine), 114.7 (2C, aromatic), 114.9 (2C, aromatic), 117.2 (C≡N), 119.5 (2C, aromatic), 129.9 (C, aromatic), 130.2 (2C, aromatic), 133.6 (C, aromatic), 140.2 (C_3a_, pyrazolopyrimidine), 149.6 (C, aromatic), 154.4 (C_2_, pyrazolopyrimidine), 156.3 (C, aromatic), 161.5 (C_5_, pyrazolopyrimidine), 161.9 (C_7_, pyrazolopyrimidine), 166.3 (C=O, amide). Anal. Calcd. (%) for C_22_H_19_N_7_O_3_ (429.43): C, 61.53; H, 4.46; N, 22.83. Found: C, 61.40; H, 4.52; N, 22.90 %.

#### 7-Amino-6-cyano-2-[(4-methoxyphenyl)amino]-5-(naphthalen-1-yl)pyrazolo[1,5-a]-pyrimidine-3-carboxamide **(7f)**

Yield: 70%, reddish-orange crystals, m.p. 252–254 °C. IR (KBr) ν_max_/cm^−1^ 3416, 3365, 3142 (NH, NH_2_), 2210 (C≡N), 1641 (C=O), 1594 (C=N), 1563 (C=C, aromatic). ^1^H NMR (DMSO-d_6_, δ ppm) 3.68 (s, 3H, OCH_3_), 6.84 (d, 2H, aromatic, *J_HH_=8.4 Hz*), 7.16 (d, 1H, aromatic, *J_HH_=8.4 Hz*), 7.29 (s, 1H, aromatic), 7.47 (s, 2H, NH_2_, D_2_O exchangeable), 7.64–7.72 (m, 3H, aromatic), 8.05 (d, 1H, aromatic, *J_HH_=7.6 Hz*), 8.21 (d, 2H, aromatic, *J_HH_=8.4 Hz*), 8.34 (d, 1H, aromatic, *J_HH_=6.9 Hz*), 8.82 (s, 2H, NH_2_, D_2_O exchangeable), 9.65 (s, 1H, NH, D_2_O exchangeable). MS m/z (%): 449 (0.39) [M^+^]. Anal. Calcd. (%) for C_25_H_19_N_7_O_2_ (449.46): C, 66.81; H, 4.26; N, 21.81. Found: C, 66.95; H, 4.21; N, 21.74 %.

### Synthesis of 3-(arylamino)-5-[(2-oxoindolin-3-ylidene)amino]-1H-pyrazole-4-carboxamides (9a,b)

A mixture of compound **1a** or **1b** (0.01 mol) with isatin **8** (0.01 mol) and triethylamine (3 drops) was refluxed in ethanol (30 mL) for 4 h. The precipitate obtained was filtered off, well-dried, and recrystallized from ethanol to give **9a,b**.

#### 5-[(2-Oxo-1,2-dihydro-3H-indol-3-ylidene)amino]-3-(phenylamino)-1H-pyrazole-4-carboxamide **(9a)**

Yield: 82%, dark brown crystals, m.p. > 300 °C. IR (KBr) ν_max_/cm^−1^ 3427, 3317, 3181 (NH, NH_2_), 1694, 1623 (C=O), 1597 (C=N), 1569 (C=C, aromatic). ^1^H NMR (DMSO-d_6_, δ ppm) 6.89–7.53 (m, 11H, aromatic and NH_2_), 9.14 (s, 1H, NH, D_2_O exchangeable), 10.95 (s, 1H, NH, D_2_O exchangeable), 12.98 (s, 1H, NH, D_2_O exchangeable). Anal. Calcd. (%) for C_18_H_14_N_6_O_2_ (346.34): C, 62.42; H, 4.07; N, 24.27. Found: C, 62.30; H, 4.14; N, 24.21 %.

#### 3-[(4-Methoxyphenyl)amino]-5-[(2-oxo-1,2-dihydro-3H-indol-3-ylidene)amino]-1H-pyrazole-4-carboxamide **(9b)**

Yield: 82%, dark green crystals, m.p. > 300 °C. IR (KBr) ν_max_/cm^−1^ 3414, 3308, 3162 (NH, NH_2_), 1688, 1623 (C=O), 1599 (C=N), 1570 (C=C, aromatic). ^1^H NMR (DMSO-d_6_, δ ppm) 3.71 (s, 3H, OCH_3_), 6.82–7.69 (m, 10H, aromatic and NH_2_), 8.94 (s, 1H, NH, D_2_O exchangeable), 10.94 (s, 1H, NH, D_2_O exchangeable), 12.92 (s, 1H, NH, D_2_O exchangeable). MS m/z (%): 376 (73.36) [M^+^]. Anal. Calcd. (%) for C_19_H_16_N_6_O_3_ (376.37): C, 60.63; H, 4.28; N, 22.33. Found: C, 60.50; H, 4.33; N, 22.27 %.

### Synthesis of 3-(arylamino)-5-(arylmethyleneamino)-1H-pyrazole-4-carboxamides (12a–j)

A mixture of compound **1a** or **1b** (0.01 mol) with an aromatic-, heteroaromatic aldehyde, or ferrocenecarboxaldehyde **11a–e** (0.01 mol) and triethylamine (four drops) was refluxed in absolute ethanol (30 mL) for 6 h. The volatile materials were removed under reduced pressure; the solid obtained was collected and recrystallized from ethanol to give **12a–j**.

#### 5-(Benzylideneamino)-3-(phenylamino)-1H-pyrazole-4-carboxamide **(12a)**

Yield: 76%, yellow crystals, m.p. 269–271 °C. IR (KBr) ν_max_/cm^−1^ 3384, 3190 (NH, NH_2_), 1653 (C=O), 1593 (C=N), 1562 (C=C, aromatic). ^1^H NMR (DMSO-d_6_, δ ppm) 6.83–7.38 (m, 4H, aromatic), 7.47 (s, 2H, NH_2_, D_2_O exchangeable), 7.56–7.96 (m, 6H, aromatic), 8.91 (s, 1H, NH, D_2_O exchangeable), 9.07 (s, 1H, –N=CH–), 12.75 (s, 1H, NH, D_2_O exchangeable). ^13^C NMR (DMSO-d_6_, δ ppm) 92.9 (C_4_, pyrazole), 116.4 (2C, aromatic), 119.7 (C, aromatic), 127.9 (2C, aromatic), 129.0 (2C, aromatic), 129.5 (2C, aromatic), 130.8 (C, aromatic), 135.3 (C, aromatic), 141.9 (C, aromatic), 148.2 (−N=CH−), 153.4 (C_3_ & C_5_, pyrazole), 167.4 (C=O, amide). Anal. Calcd. (%) for C_17_H_15_N_5_O (305.33): C, 66.87; H, 4.95; N, 22.94. Found: C, 66.94; H, 4.87; N, 22.89 %.

#### 5-[(4-Methoxybenzylidene)amino]-3-(phenylamino)-1H-pyrazole-4-carboxamide **(12b)**

Yield: 65%, yellow crystals, m.p. 260 °C. IR (KBr) ν_max_/cm^−1^ 3399, 3317, 3203 (NH, NH_2_), 1648 (C=O), 1596 (C=N), 1556 (C=C, aromatic). ^1^H NMR (DMSO-d_6_, δ ppm) 3.85 (s, 3H, OCH_3_), 6.83 (t, 1H, aromatic), 7.12 (d, 2H, aromatic, *J_HH_=*7.6 Hz), 7.25–7.38 (m, 4H, aromatic), 7.52 (s, 2H, NH_2_, D_2_O exchangeable), 7.94 (d, 2H, aromatic, *J_HH_=*7.6 Hz), 8.81 (s, 1H, NH, D_2_O exchangeable), 9.10 (s, 1H, –N=CH–), 12.62 (s, 1H, NH, D_2_O exchangeable). ^13^C NMR (DMSO-d_6_, δ ppm) 56.1 (−OCH_3_), 93.2 (C_4_, pyrazole), 115.2 (2C, aromatic), 116.6 (2C, aromatic), 119.9 (C, aromatic), 128.1 (C, aromatic), 129.5 (2C, aromatic), 131.9 (2C, aromatic), 142.3 (C, aromatic), 147.8 (−N=CH−), 153.1 (C_3_ & C_5_, pyrazole), 163.7 (C, aromatic), 166.9 (C=O, amide). MS m/z (%): 335 (100) [M^+^]. Anal. Calcd. (%) for C_18_H_17_N_5_O_2_ (335.36): C, 64.47; H, 5.11; N, 20.88. Found: C, 64.57; H, 5.06; N, 20.97 %.

#### 5-[(Naphthalen-1-ylmethylidene)amino]-3-(phenylamino)-1H-pyrazole-4-carboxamide **(12c)**

Yield: 76%, buff crystals, m.p. 263–265 °C. IR (KBr) ν_max_/cm^−1^ 3357, 3144 (NH, NH_2_), 1645 (C=O), 1592 (C=N), 1558 (C=C, aromatic). ^1^H NMR (DMSO-d_6_, δ ppm) 6.83–8.83 (m, 14H, aromatic and NH_2_), 9.10 (s, 1H, –N=CH–), 9.66 (s, 1H, NH, D_2_O exchangeable), 12.89 (s, 1H, NH, D_2_O exchangeable). Anal. Calcd. (%) for C_21_H_17_N_5_O (355.39): C, 70.97; H, 4.82; N, 19.71. Found: C, 70.88; H, 4.88; N, 19.79 %.

#### 5-{[(5-Methylfuran-2-yl)methylidene]amino}-3-(phenylamino)-1H-pyrazole-4-carboxamide **(12d)**

Yield: 80%, yellow crystals, m.p. 235–238 °C. IR (KBr) ν_max_/cm^−1^ 3367, 3181 (NH, NH_2_), 1651 (C=O), 1594 (C=N), 1565 (C=C, aromatic). ^1^H NMR (DMSO-d_6_, δ ppm) 2.40 (s, 3H, CH_3_), 6.43–7.35 (m, 7H, aromatic, furan H-4 and furan H-3), 7.48 (s, 2H, NH_2_, D_2_O exchangeable), 8.56 (s, 1H, NH, D_2_O exchangeable), 9.04 (s, 1H, –N=CH–), 12.59 (s, 1H, NH, D_2_O exchangeable). ^13^C NMR (DMSO-d_6_, δ ppm) 14.3 (−CH_3_), 93.8 (C_4_, pyrazole), 110.9 (C_4_, furan), 116.6 (C_3_, furan), 120.0 (2C, aromatic), 123.7 (C, aromatic), 129.5 (2C, aromatic), 141.9 (C, aromatic), 147.4 (−N=CH−), 149.6 (C_2_, furan), 150.1 (C_5_, furan), 153.4 (C_3_ & C_5_, pyrazole), 166.8 (C=O, amide). MS m/z (%): 309 (21.84) [M^+^]. Anal. Calcd. (%) for C_16_H_15_N_5_O_2_ (309.32): C, 62.13; H, 4.89; N, 22.64. Found: C, 62.00; H, 4.94; N, 22.55 %.

#### 5-[(Ferrocene-1-ylmethylidene)amino]-3-(phenylamino)-1H-pyrazole-4-carboxamide **(12e)**

Yield: 83%, red-pirck crystals, m.p. 255 °C. IR (KBr) ν_max_/cm^−1^ 3359, 3168 (NH, NH_2_), 1660 (C=O), 1588 (C=N), 1563 (C=C, aromatic). ^1^H NMR (DMSO-d_6_, δ ppm) 4.29 (s, 5H, C_5_H_5_, ferrocene ring protons), 4.68 (s, 2H, C_5_H_4_, ferrocene ring protons), 4.88 (s, 2H, C_5_H_4_, ferrocene ring protons), 6.79–7.28 (m, 5H, aromatic), 7.50 (s, 2H, NH_2_, D_2_O exchangeable), 8.72 (s, 1H, NH, D_2_O exchangeable), 9.03 (s, 1H, –N=CH–), 12.55 (s, 1H, NH, D_2_O exchangeable). ^13^C NMR (DMSO-d_6_, δ ppm) 70.2 (5C, ferrocene ring), 73.3 (4C, ferrocenyl ring), 79.1 (C, ferrocenyl ring), 92.8 (C_4_, pyrazole), 116.6 (2C, aromatic), 119.8 (C, aromatic), 129.5 (2C, aromatic), 142.0 (C, aromatic), 148.3 (−N=CH−), 153.2 (C_3_ & C_5_, pyrazole), 167.0 (C=O, amide). MS m/z (%): 413 (100%) [M^+^]. Anal. Calcd. (%) for C_21_H_19_FeN_5_O (413.25): C, 61.03; H, 4.63; N, 16.95. Found: C, 60.90; H, 4.69; N, 17.05 %.

#### 5-(Benzylideneamino)-3-[(4-methoxyphenyl)amino]-1H-pyrazole-4-carboxamide **(12f)**

Yield: 79%, orange crystals, m.p. 260–262 °C. IR (KBr) ν_max_/cm^−1^ 3396, 3181 (NH, NH_2_), 1655 (C=O), 1594 (C=N), 1565 (C=C, aromatic). ^1^H NMR (DMSO-d_6_, δ ppm) 3.68 (s, 3H, OCH_3_), 6.85–7.37 (m, 5H, aromatic), 7.55 (s, 2H, NH_2_, D_2_O exchangeable), 7.59 (d, 2H, aromatic, *J_HH_=*7.6 Hz), 7.95 (d, 2H, aromatic, *J_HH_=*7.6 Hz), 8.82 (s, 1H, NH, D_2_O exchangeable), 8.91 (s, 1H, –N=CH–), 12.58 (s, 1H, NH, D_2_O exchangeable). ^13^C NMR (DMSO-d_6_, δ ppm) 55.6 (−OCH_3_), 92.8 (C_4_, pyrazole), 114.9 (2C, aromatic), 118.2 (2C, aromatic), 128.2 (2C, aromatic), 129.5 (2C, aromatic), 131.3 (C, aromatic), 133.6 (C, aromatic), 135.1 (C, aromatic), 135.7 (C, aromatic), 148.1 (−N=CH−), 153.3 (C_3_ & C_5_, pyrazole), 167.3 (C=O, amide). Anal. Calcd. (%) for C_18_H_17_N_5_O_2_ (335.36): C, 64.47; H, 5.11; N, 20.88. Found: C, 64.56; H, 5.06; N, 20.75 %.

#### 5-[(4-Methoxybenzylidene)amino]-3-[(4-methoxyphenyl)amino]-1H-pyrazole-4-carboxamide **(12g)**

Yield: 81%, yellow crystals, m.p. >300 °C. IR (KBr) ν_max_/cm^−1^ 3395, 3194 (NH, NH_2_), 1651 (C=O), 1598 (C=N), 1511 (C=C, aromatic). ^1^H NMR (DMSO-d_6_, δ ppm) 3.68 (s, 3H, OCH_3_), 3.83 (s, 3H, OCH_3_), 6.83–7.26 (m, 6H, aromatic), 7.43 (s, 2H, NH_2_, D_2_O exchangeable), 7.91 (d, 2H, aromatic, *J_HH_=*8.4 Hz), 8.80 (s, 1H, NH, D_2_O exchangeable), 8.82 (s, 1H, –N=CH–), 12.50 (s, 1H, NH, D_2_O exchangeable). ^13^C NMR (DMSO-d_6_, δ ppm) 55.6 (−2OCH_3_), 92.7 (C_4_, pyrazole), 114.9 (2C, aromatic), 115.4 (2C, aromatic), 117.8 (2C, aromatic), 128.2 (C, aromatic), 131.6 (2C, aromatic), 133.5 (C, aromatic), 135.8 (C, aromatic), 147.9 (−N=CH−), 153.2 (C_3_ & C_5_, pyrazole), 163.9 (C, aromatic), 166.9 (C=O, amide). Anal. Calcd. (%) for C_19_H_19_N_5_O_3_ (365.39): C, 62.46; H, 5.24; N, 19.17. Found: C, 62.58; H, 5.18; N, 19.10 %.

#### 3-[(4-Methoxyphenyl)amino]-5-[(naphthalen-1-ylmethylidene)amino]-1H-pyrazole-4-carboxamide **(12h)**

Yield: 68%, reddish-orange crystals, m.p. 250–252 °C. IR (KBr) ν_max_/cm^−1^ 3365, 3149 (NH, NH_2_), 1643 (C=O), 1595 (C=N), 1563 (C=C, aromatic). ^1^H NMR (DMSO-d_6_, δ ppm) 3.69 (s, 3H, OCH_3_), 6.86–8.32 (m, 13H, aromatic and NH_2_), 8.85 (s, 1H, NH, D_2_O exchangeable), 9.66 (s, 1H, –N=CH–), 12.78 (s, 1H, NH, D_2_O exchangeable). ^13^C NMR (DMSO-d_6_, δ ppm) 55.7 (−OCH_3_), 93.1 (C_4_, pyrazole), 114.9 (2C, aromatic), 118.3 (2C, aromatic), 123.7 (C, aromatic), 126.2 (C, aromatic), 127.1 (2C, aromatic), 128.4 (C, aromatic), 129.6 (2C, aromatic), 130.6 (2C, aromatic), 131.8 (2C, aromatic), 134.0 (C, aromatic), 148.6 (−N=CH−), 153.2 (C_3_ & C_5_, pyrazole), 166.9 (C=O, amide). Anal. Calcd. (%) for C_22_H_19_N_5_O_2_ (385.42): C, 68.56; H, 4.97; N, 18.17. Found: C, 68.41; H, 5.04; N, 18.07 %.

#### 3-[(4-Methoxyphenyl)amino]-5-{[(5-methylfuran-2-yl)methylidene]amino}-1H-pyrazole-4-carboxamide**(12i)**

Yield: 70%, buff crystals, m.p. 232–234 °C. IR (KBr) ν_max_/cm^−1^ 3346, 3157 (NH, NH_2_), 1652 (C=O), 1588 (C=N), 1512 (C=C, aromatic). ^1^H NMR (DMSO-d_6_, δ ppm) 2.39 (s, 3H, CH_3_), 3.68 (s, 3H, OCH_3_), 6.42–7.35 (m, 6H, aromatic, furan H-4 and furan H-3), 7.42 (s, 2H, NH_2_, D_2_O exchangeable), 8.56 (s, 1H, NH, D_2_O exchangeable), 8.78 (s, 1H, –N=CH–), 12.45 (s, 1H, NH, D_2_O exchangeable). ^13^C NMR (DMSO-d_6_, δ ppm) 14.2 (−CH_3_), 55.6 (−OCH_3_), 93.6 (C_4_, pyrazole), 110.8 (C_4_, furan), 116.3 (C_3_, furan), 120.1 (2C, aromatic), 123.7 (2C, aromatic), 132.2 (C, aromatic), 147.1 (−N=CH−), 149.0 (C_2_, furan), 150.5 (C_5_, furan), 151.4 (C, aromatic), 153.0 (C_3_ & C_5_, pyrazole), 167.1 (C=O, amide). Anal. Calcd. (%) for C_17_H_17_N_5_O_3_ (339.35): C, 60.17; H, 5.05; N, 20.64. Found: C, 60.06; H, 5.11; N, 20.73 %.

#### 5-[(Ferrocene-1-ylmethylidene)amino]-3-[(4-methoxyphenyl)amino]-1H-pyrazole-4-carboxamide **(12j)**

Yield: 76%, brown crystals, m.p. 230–232 °C. IR (KBr) ν_max_/cm^−1^ 3378, 3202 (NH, NH_2_), 1631 (C=O), 1589 (C=N), 1562 (C=C, aromatic). ^1^H NMR (DMSO-d_6_, δ ppm) 3.67 (s, 3H, OCH_3_), 4.28 (s, 5H, C_5_H_5_, ferrocene ring protons), 4.67 (s, 2H, C_5_H_4_, ferrocene ring protons), 4.87 (s, 2H, C_5_H_4_, ferrocene ring protons), 6.83–7.22 (m, 4H, aromatic), 7.43 (s, 2H, NH_2_, D_2_O exchangeable), 8.70 (s, 1H, NH, D_2_O exchangeable), 8.78 (s, 1H, –N=CH–), 12.49 (s, 1H, NH, D_2_O exchangeable). ^13^C NMR (DMSO-d_6_, δ ppm) 55.7 (−OCH_3_), 70.1 (5C, ferrocene ring), 73.3 (4C, ferrocenyl ring), 79.1 (C, ferrocenyl ring), 92.6 (C_4_, pyrazole), 114.8 (2C, aromatic), 117.7 (2C, aromatic), 133.8 (C, aromatic), 135.8 (C, aromatic), 148.4 (−N=CH−), 153.2 (C_3_ & C_5_, pyrazole), 167.1 (C=O, amide). MS m/z (%): 443 (10.10%) [M^+^]. Anal. Calcd. (%) for C_22_H_21_FeN_5_O_2_ (443.28): C, 59.61; H, 4.78; N, 15.80. Found: C, 59.71; H, 4.71; N, 15.70 %.

### Biological experiments

#### In vitro antitumor screening

The tested compounds were subjected to *in vitro* disease-oriented primary antitumor screening. The different cell lines of tumor cell lines were utilized. The human tumor cell lines of the cancer screening panel were grown in RPMI 1640 medium containing 5% fetal bovine serum and 2 mM L-glutamine. For a typical screening experiment, cells were inoculated into 96-well microtiter plates in 100 mL at plating densities ranging from 5000 to 40,000 cells/well depending on the doubling time of individual cell lines. After cell inoculation, the microtiter plates were incubated at 37°C, 5% CO_2_, 95% air, and 100% relative humidity for 24 h prior to the addition of the experimental drugs. After 24 h, two plates of each cell line were fixed *in situ* with TCA, to represent a measurement of the cell population for each cell line at the time of drug addition. Experimental drugs were solubilized in DMSO at 400-fold of the desired final maximum test concentration and stored frozen prior to use. At the time of drug addition, an aliquot of frozen concentrate was thawed and diluted to twice the desired final maximum test concentration with complete medium containing 50 mg/ml Gentamicin. Additional four 10-fold or 1/2 log serial dilutions were made to provide a total of five drug concentrations plus the control. Aliquots of 100 mL of these different drug dilutions were added to the appropriate microtiter wells already containing 100 mL of medium, resulting in the required final drug concentrations. Following drug addition, the plates were incubated for an additional 48 h at 37°C, 5% CO_2_, 95% air, and 100% relative humidity. For adherent cells, the assay was terminated by the addition of cold TCA. Cells were fixed *in situ* by the gentle addition of 50 mL of cold 50% (w/v) TCA (final concentration, 10% TCA) and incubated for 60 min at 4°C. The supernatant layer was discarded, and the plates were washed five times with tap water and air-dried. Sulforhodamine B (SRB) solution (100 mL) at 0.4% (w/v) in 1% acetic acid was added to each well, and plates were incubated for 10 min at room temperature. After staining, unbound dye was removed by washing five times with 1% acetic acid and the plates were air-dried. Bound stain was subsequently solubilized with 10 mM trizma base, and the absorbance was read on an automated plate reader at a wavelength of 515 nm.

For suspension cells, the methodology was the same except that the assay was terminated by fixing settled cells at the bottom of the wells by gently adding 50 mL of 80% TCA (final concentration, 16% TCA). The parameter used here was GI_50,_ which is the log10 concentration at which PG is 50, and was calculated for each cell line [19–21].

## Conclusion

In conclusion, 5-amino-1*H*-pyrazole-4-carboxamide derivatives **1a,b** were used as starting materials for the synthesis of some new pyrazolo[1,5-*a*]pyrimidine derivatives and Schiff bases. The new synthesized compounds were characterized by analytical and spectroscopic data. Some selected new compounds were screened for their potential antitumor activities. The results of the cytotoxicity for the tested compounds against different human cancer cell lines indicated that most of them exhibit a high cytotoxicity at very low concentrations in comparison with the reference drugs used, and pyrazolo[1,5-*a*]-pyrimidine derivatives **4a**, **7e,** and **7f** were found to have the most potent growth inhibitory activity against MCF7, ovarial carcinoma (SK OV-3), leukemia (K562), and HeLa (cervical) human tumor cell lines. On the other hand, Schiff bases **12b–e** and **12j** were found to be the most potent against cervical carcinoma (KB), CNS cancer (SF-268), non-small cell lung cancer (NCl H460), colonadenocarcinoma (RKOP 27), anti-leukemia (HL60, U937), melanoma (SK-MEL-28), neuroblastoma (GOTO, NB-1), HT1080 (fibrosarcoma), and HepG2 (liver) human tumor cell lines.

## Figures and Tables

**Sch. 1.**
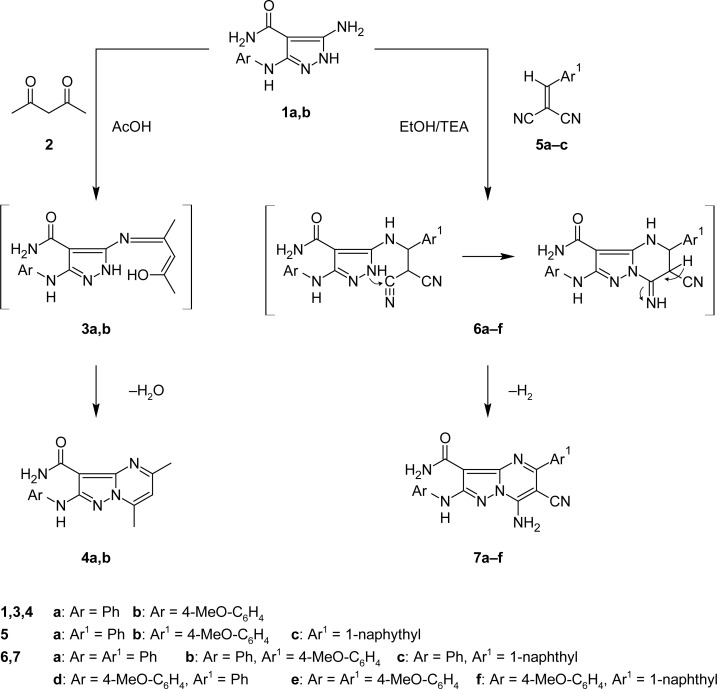
Synthesis of pyrazolo[1,5-*a*]pyrimidine derivatives

**Sch. 2.**
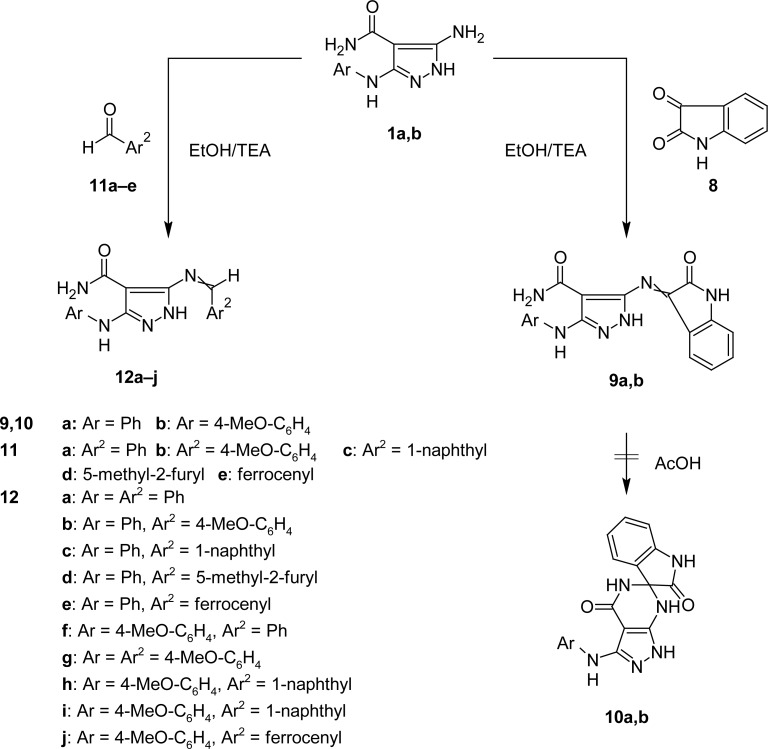
Reaction of 1*H*-pyrazolo-4-carboxamide derivatives with isatin and some selected aldehydes.

**Tab. 1.** The cytotoxicity of the tested compounds on the MCF-7 tumor cell line.

**Cpd.**	**IC_50_^a^ μM Tumor cell growth inhibition (%)**
**4a**	122.9
**7f**	0.085*
**12d**	280.0
**12e**	28.48
**12j**	9.294
**Doxorubicin**	96.41

a The concentration required for 50% inhibition of cell growth.

* The most potent compound.

**Tab. 2.** The cytotoxicity of synthesized compounds was determined by using the MTT assay on different human cancer cell lines

**Cpd.**	**IC_50_^a^ nM Tumor cell growth inhibition (%)**				

	**KB**	**SK OV-3**	**SF-268**	**NCI H460**	**RKOP27**
**4a**	7.70	6.50	0.90	0.80	6.00
**4b**	4.88	6.73	7.70	0.60	4.00
**7e**	0.67	0.30*	7.00	0.70	8.00
**7f**	4.50	7.80	0.60	0.70	7.00
**9b**	8.50	8.70	8.00	0.89	8.00
**12b**	0.65	0.44	8.00	0.77	5.00
**12c**	0.76	4.40	3.00	0.60	0.50*
**12d**	0.78	3.30	0.65	0.60	3.00
**12e**	0.54*	0.32	0.30*	6.60	0.60
**12f**	0.67	0.90	6.00	0.70	7.00
**12h**	4.40	2.20	6.00	7.00	0.70
**12j**	0.77	5.60	0.44	0.50*	4.00
**Fluorouracil**	4.46	–	–	–	–
**Doxorubicin**	–	4.16	–	–	–
**Cytarabine**	–	–	7.68	–	–
**Gemcitabine HCl**	–	–	–	2.13	–
**Capecitabine**	–	–	–	–	4.33

a The concentration required for 50% inhibition of cell growth.

* The most potent compound.

**Tab. 3.** The cytotoxicity of synthesized compounds was determined by using the MTT assay on different human cancer cell lines

**Cpd.**	**IC_50_^a^ nM Tumor cell growth inhibition (%)**					

	**Leukemia**			**Melanoma**	**Neuroblastoma**	

	**HL60**	**U937**	**K562**	**SK-MEL-28**	**GOTO**	**NB-1**
**4a**	0.57	6.60	0.78	5.40	0.46	5.80
**4b**	7.50	7.80	0.50	7.90	5.60	6.43
**7e**	0.50	0.60	8.00	7.00	0.69	0.65
**7f**	3.57	55.0	0.17*	66.0	6.00	5.29
**9b**	5.30	7.00	4.30	6.00	5.00	2.59
**12b**	0.88	5.40	0.70	3.20*	0.60	5.45
**12c**	0.78	7.70	0.65	7.30	0.67	6.53
**12d**	0.43*	0.09*	4.00	8.40	0.45*	0.64*
**12e**	0.66	6.40	0.43	6.80	0.67	3.47
**12f**	0.55	0.50	6.50	66.0	0.60	0.69
**12h**	5.50	4.00	0.79	7.00	0.79	5.54
**12j**	5.60	8.80	7.80	6.90	5.47	4.27
**Doxorubicin**	1.13	4.45	6.66	–	4.73	5.15
**Aldesleukin**	–	–	–	3.45	–	–

a The concentration required for 50% inhibition of cell growth.

* The most potent compound.

**Tab. 4.** The cytotoxicity of synthesized compounds was determined by using the MTT assay on different human cancer cell lines

**Cpd.**	**IC_50_^a^ nM Tumor cell growth inhibition (%)**		

	**HeLa (cervical)**	**HT1080 (fibrosarcoma)**	**HepG2 (liver)**
**4a**	0.36*	0.96	8.70
**4b**	7.59	7.63	8.47
**7e**	0.87	0.59	0.96
**7f**	3.96	3.60	4.45
**9b**	9.50	8.50	8.00
**12b**	0.42	0.84	7.80
**12c**	0.73	8.97	0.66
**12d**	0.80	0.54*	0.35
**12e**	7.60	8.48	0.09*
**12f**	0.90	0.64	0.86
**12h**	0.75	0.65	0.99
**12j**	8.48	0.78	0.62
**Tamoxifen**	0.11	1.16	1.31

a The concentration required for 50% inhibition of cell growth.

* The most potent compound.

## References

[1] Quintela JM, Peinador C, Moreira MJ, Alfonso A, Botana LM, Riguera R (2001). Pyrazolopyrimidines: synthesis, effect on histamine release from rat peritoneal mast cells and cytotoxic activity. Eur J Med Chem.

[2] Popovici-Muller J, Jr GWS, Rosner KE, Deng Y, Wang T, Curran PJ, Brown MA, Siddiqui MA, Cooper AB, Duca J, Cable M, Girijavallabhan V (2009). Pyrazolo[1,5-*a*]pyrimidine-based inhibitors of HCV polymerase. Bioorg Med Chem Lett.

[3] Dwyer MP, Paruch K, Labroli M, Alvarez C, Keertikar KM, Poker C, Rossman R, Fischmann TO, Duca JS, Madison V, Parry D, Davis N, Seghezzi W, Wiswell D, Guzi TJ (2011). Discovery of pyrazolo[1,5-*a*]pyrimidine-based CHK1 inhibitors: A template-based approach-Part 1. Bioorg Med Chem Lett.

[4] Williamson DS, Parratt MJ, Bower JF, Moore JD, Richardson CM, Dokurno P, Cansfield AD, Francis GL, Hebdon RJ, Howes R, Jackson PS, Lockie AM, Murray JB, Nunns CL, Powles J, Robertson A, Surgenor AE, Torrance CJ (2005). Structure-guided design of pyrazolo[1,5-*a*]pyrimidines as inhibitors of human cyclin-dependent kinase2. Bioorg Med Chem Lett.

[5] Mukaiyama H, Nishimura T, Kobayashi S, Komatsu Y, Kikuchi S, Ozawa T, Kamada N, Ohnota H (2008). Novel pyrazolo[1,5-*a*]pyrimidines as c-Src kinase inhibitors that reduce *I**_Kr_* channel blockade. Bioorg Med Chem.

[6] Aggarwal R, Sumran G, Garg N, Aggarwal A (2011). A regioselective synthesis of some new pyrazol-1′-ylpyrazolo[1,5-*a*]pyrimidines in aqueous medium and their evaluation as antimicrobial agents. Eur J Med Chem.

[7] Abd El Razik HA, Abdel Wahab AE (2011). Synthesis and biological evaluation of some novel fused pyrazolopyrimidines as potential anticancer and antimicrobial agents. Arch Pharm.

[8] Alcaro S, Artese A, Botta M, Zizzari AT, Orallo F, Ortuso F, Schenone S, Brullo C, Yáñez M (2010). Hit identification and biological evaluation of anticancer pyrazolopyrimidines endowed with anti-inflammatory activity. ChemMedChem.

[9] Golcu A, Tumer M, Demirelli H, Wheatley RA (2005). Cd(II) and Cu(II) complexes of polydentate Schiff base ligands: synthesis, characterization, properties and biological activity. Inorg Chim Acta.

[10] Chen A, Taguchi T, Aoyama S, Sugiura M, Haruna M, Wang M, Miwa I (2003). Antioxidant activity of a schiff base of pyridoxal and aminoguanidine. Free Radic Biol Med.

[11] Li YF, Liu ZQ (2011). Ferrocenyl Schiff base as novel antioxidant to protect DNA against the oxidation damage. Eur J Pharm Sci.

[12] Qin DD, Yang ZY, Zhang FH, Du B, Wang P, Li TR (2010). Evaluation of the antioxidant, DNA interaction and tumor cell cytotoxicity activities of Copper(II) complexes with Paeonol Schiff-base. Inorg Chem Commun.

[13] Ghorab MM, Shaaban MA, Refaat HM, Heiba HI, Ibrahim SS (2012). Anticancer and radiosensitizing evaluation of some new pyranothiazole-Schiff bases bearing the biologically active sulfonamide moiety. Eur J Med Chem.

[14] Hu GQ, Wu XK, Wang GQ, Duan NN, Wen XY, Cao TY, Jun Y, Wei W, Xie SQ, Huang WL (2012). Synthesis and antitumor and antibacterial evaluation of fluoro-quinolone derivatives (III): Mono- and bis-Schiff-bases. Chin Chem Lett.

[15] Kraicheva I, Tsacheva I, Vodenicharova E, Tashev E, Tosheva T, Kril A, Topashka-Ancheva M, Iliev I, Gerasimova Ts, Troev K (2012). Synthesis, antiproliferative activity and genotoxicity of novel anthracene-containing aminophosphonates and a new anthracene-derived Schiff base. Bioorg Med Chem.

[16] Elgemeie GH, Elsayed SH, Hassan AS (2008). Direct route to a new class of acrylamide thioglycosides and their conversions to pyrazole derivatives. Synth Commun.

[17] Ghozlan SAS, Hassanien AA (2002). β-Amino-β-(pyrid-4-yl)acrylonitrile in heterocyclic synthesis: synthesis of some new pyridine, pyridone, pyrazole, thiophene, fused pyrimidine and triazine derivatives. Tetrahedron.

[18] Pinto-Garcia L, Efferth T, Torres A, Hoheisel JD, Youns M (2010). Berberine inhibits cell growth and mediates caspase-independent cell death in human pancreatic cancer cells. Planta Med.

[19] Grever MR, Schepartz SA, Chabner BA (1992). The National Cancer Institute: Cancer Drug Discovery and Development Program. Sem Oncol.

[20] Boyd MR, Paull KD (1995). Some practical considerations and applications of the national cancer institute*in vitro* anticancer drug discovery screen. Drug Dev Res.

[21] Monks A, Scudiero D, Skehan P, Shoemaker R, Paull K, Vistica D, Hose C, Langley J, Cronise P, Vaigro-Wolff A, Gray-Goodrich M, Campbell H, Mayo J, Boyd M (1991). Feasibility of a High-Flux Anticancer Drug Screen Using a Diverse Panel of Cultured Human Tumor Cell Lines. J Natl Cancer Inst.

